# Pyorrhœa Alveolaris.—A Case in Practice

**Published:** 1878-09

**Authors:** W. H. Eames


### ARTICLE VI.

### *Pyorrhœa Alveolaris.—A Case in Practice.*

Mrs. M———, thirty-five years; nervo-sanguine temperament; general health good: called for consultation in regard to her teeth and gums, which were causing her much much pain and uneasiness.

History of the Case.—She had been troubled for several months with an uneasy feeling, sometimes amounting to pain in the gums and teeth of both the upper and lower jaw. The teeth would at times appear elongated; become quite loose and painful, especially to the touch. The gums swollen and tender, bleeding readily, so as to interfere with her customary use of the brush. Was troubled with a disagreeable taste, and fetor of the breath, especially on rising in the morning. Had received some treatment, consisting of mouth washes, hypodermic injections of aromatic sulphuric acid in the borders of the gums, but with no apparent benefit. Through the advice of her dentist she had recently had the first and second left lower molars which, were very loose and painful, extracted, with the hope of relieving her of pain, and of arresting disease. The third molar was now troubling her as the others had, and she desired to know if anything could be done for her relief without the loss of this tooth.

On examination, I found the teeth, which evidently had been well cared for, all more or less loose, and painful on percussion. This was especially the case with the tooth complained of—the third left lower molar—and the molars and bicuspids of the opposite side; the central incisors of the lower jaw, and the left central and lateral of the upper, and the molar and bicuspids on the right side, upper.

The gums were of a dark livid color; lax, and very much thickened, especially at the gingival border, which was deeply festered. Being deprived of a part of the epithelial structure, the surface had a polished look, and bled easily; at some points, as at the anterior border of the incisors, the gum was entirely denuded of its epithelium and the surface had become granulous, covered with fleshy buds, small nippled vegetations, bathed by a peculiar oozing, as frequently seen on the granulating surface of a wound. The characteristics of fungous gingivitis. An examination with an exploring instrument, which could be readily passed underneath the gum nearly to the apex of the roots of several of the teeth, revealed a ring of hard, nodulated, rough, dark brown deposit on the roots of the teeth beneath the gum; this deposit was found to reach nearly to the border of the alveoli, from the ulcerating surfaces of which pus was constantly being thrown off. We have here a well marked case —as I understand it, of what has been designated as "Riggs Disease," or Pyorrhœa Alveolaris. It is in fact, a case of ulceration of the alveolar border, induced or kept up by this ring of deposit around the necks of the teeth. The etiology of the case is at present somewhat obscure, and has not, we believe, been very definitely settled.

There are several constitutional disturbances which might induce ulceration of this bony tissue, and when once set up, the rough deposit soon takes place, which in turn serves to maintain the ulcerative process, and consequent loss of the alveoli; thus the first step in the treatment is clearly indicated, viz:—the perfect removal of this deposit from the necks of the teeth. With instruments similar to those suggested by Dr. Riggs, we removed as perfectly as possible, all the deposit at the first sitting. Chloride of zinc in crystal was applied to the ulcerating surfaces by passing it under the gum around the teeth on a little cotton, wound on a broach. Tincture of Iodine, full strength, was used as a topical dressing; a tonic and astringent mouth wash with potassium chlorate, was recommended to be used after each meal, and at night, with as thorough use of the brush as the condition of the gums would allow. The first application of the zinc gave little or no pain. On the second day, the teeth were again thoroughly examined for any remaining particles of deposit, and the zinc and iodine again used; some pain followed this application, lasting but a few moments. This treatment was repeated daily for six days, omitted on the seventh and renewed on the eighth and ninth, when the patient left the city. The improvement following this treatment was very marked; the gums had assumed a normal color and appearance, the teeth ceased to be painful on percussion, and had become firmer as the tonicity of the peridental membrane was restored; hemorrhage from the gums, and the secretion of pus from the ulcerating borders of the alveoli had ceased almost entirely. The disagreeable fetor, one of the worst features in this case, had also disappeared. What the final result of the case has been, we cannot say, as the patient has not been heard from since she left the city, but we feel confident, judging from the result following this treatment in various similar cases, that if thoroughly carried out in this case, the disease has not only been arrested, but the hard and soft part alike restored to a healthy normal condition. Our experience with the chloride of zinc in these cases has been much more satisfactory than other treatment.—*Dr. Eames.*—*Missouri Dental Journal.*

